# Integrating field and laboratory approaches for ripple development in mixed sand–clay–EPS

**DOI:** 10.1111/sed.12611

**Published:** 2019-06-05

**Authors:** Jaco H. Baas, Megan L. Baker, Jonathan Malarkey, Sarah J. Bass, Andrew J. Manning, Julie A. Hope, Jeffrey Peakall, Ian D. Lichtman, Leiping Ye, Alan G. Davies, Daniel R. Parsons, David M. Paterson, Peter D. Thorne

**Affiliations:** ^1^ School of Ocean Sciences Bangor University Menai Bridge Isle of Anglesey LL59 5AB UK; ^2^ Department of Geography, Environment and Earth Sciences University of Hull Hull HU6 7RX UK; ^3^ School of Marine Science and Engineering Plymouth University Plymouth PL4 8AA UK; ^4^ HR Wallingford Howbery Park Wallingford OX10 8BA UK; ^5^ Institute of Marine Science University of Auckland Private Bag 92019 Auckland New Zealand; ^6^ School of Earth and Environment University of Leeds Leeds LS2 9JT UK; ^7^ National Oceanography Centre Joseph Proudman Building, 6 Brownlow Street Liverpool L3 5DA UK; ^8^ Department of Civil and Environmental Engineering Center for Applied Coastal Research University of Delaware Newark DE 19716 USA; ^9^ School of Biology Scottish Oceans Institute University of St Andrews St. Andrews KY16 8LB UK

**Keywords:** Cohesion, current ripples, estuary, intertidal flat, mixed sand–clay–EPS

## Abstract

The shape and size of sedimentary bedforms play a key role in the reconstruction of sedimentary processes in modern and ancient environments. Recent laboratory experiments have shown that bedforms in mixed sand–clay develop at a slower rate and often have smaller heights and wavelengths than equivalent bedforms in pure sand. This effect is generally attributed to cohesive forces that can be of physical origin, caused by electrostatic forces of attraction between clay minerals, and of biological origin, caused by ‘sticky’ extracellular polymeric substances (EPS) produced by micro‐organisms, such as microalgae (microphytobenthos) and bacteria. The present study demonstrates, for the first time, that these laboratory experiments are a suitable analogue for current ripples formed by tidal currents on a natural mixed sand–mud–EPS intertidal flat in a macrotidal estuary. Integrated hydrodynamic and bed morphological measurements, collected during a spring tide under weak wave conditions near Hilbre Island (Dee Estuary, north‐west England, UK), reveal a statistically significant decrease in current ripple wavelength for progressively higher bed mud and EPS contents, and a concurrent change from three‐dimensional linguoid to two‐dimensional straight‐crested ripple planform morphology. These results agree well with observations in laboratory flumes, but the rate of decrease of ripple wavelength as mud content increased was found to be substantially greater for the field than the laboratory. Since the formation of ripples under natural conditions is inherently more complex than in the laboratory, four additional factors that might affect current ripple development in estuaries, but which were not accounted for in laboratory experiments, were explored. These were current forcing, clay type, pore water salinity and bed EPS content. These data illustrate that clay type alone cannot explain the difference in the rate of decrease in ripple wavelength, because the bed clay contents were too low for clay type to have had a measurable effect on bedform development. Accounting for the difference in current forcing between the field and experiments, and therefore the relative stage of development with respect to equilibrium ripples, increases the difference between the ripple wavelengths. The presence of strongly cohesive EPS in the current ripples on the natural intertidal flat might explain the majority of the difference in the rate of decrease in ripple wavelength between the field and the laboratory. The effect of pore water salinity on the rate of bedform development cannot be quantified at present, but salinity is postulated herein to have had a smaller influence on the ripple wavelength than bed EPS content. The common presence of clay and EPS in many aqueous sedimentary environments implies that a re‐assessment of the role of current ripples and their primary current lamination in predicting and reconstructing flow regimes is necessary, and that models that are valid for pure sand are an inappropriate descriptor for more complex mixed sediment. This study proposes that this re‐assessment is necessary at all bed clay contents above 3%.

## Introduction

In estuaries, the interaction of river flows, tides and waves leads to more complex particle movement and resulting spatiotemporal distribution of deposits than in many other environments (Dalrymple & Choi, 2007; Van den Berg *et al*., 2007). In particular, the common presence of sand mixed with cohesive clay and non‐cohesive silt – with silt and clay collectively referred to as mud herein – renders the reconstruction of sedimentary processes from bedforms and primary current lamination in estuarine sedimentary facies a challenge, because cohesive forces can have a large influence on the erosion, transport and deposition of sediment (Mehta, 2013; Chen *et al*., 2017). Even small volumes of mud‐sized particles in sand – well within ‘clean sand’ and ‘mature sandstone’ (arenite), defined as sand with <25% mud by Shepard (1954) and <10% to 15% mud by Folk (1951) and Dott (1964) – are able to bind sediment particles via electrostatic van der Waals forces. This binding increases the threshold stress for sediment entrainment from the seabed, promotes flocculation of suspended sediment, and changes depositional properties, compared to non‐cohesive silt and sand (Mehta, 2013). In addition to this physical cohesion, estuaries are also major sites of primary and secondary production, which promotes the development of biological cohesion by organic molecules produced through biological activity (for example, extracellular polymeric substances, EPS), acting to bind particles through electrostatic interactions, hydrogen bond formation and cation effects (Underwood & Paterson, 2003). Extracellular polymeric substances are produced in sediment by microphytobenthos; chiefly diatoms and cyanobacteria (Underwood & Paterson, 2003), and bacteria.

At present, cohesion is not sufficiently well‐incorporated either into engineering models for estuarine sediment transport or into geological facies models of estuaries (Le Hir *et al*., 2007; Wang & Sturm, 2016). Recent experimental work has shown that further fundamental physical sedimentological research is needed to close the knowledge gap with non‐cohesive sedimentary dynamics (Baas *et al*., 2013, 2016; Malarkey *et al*., 2015; Schindler *et al*., 2015; Parsons *et al*., 2016). These laboratory studies have highlighted that sedimentary bedforms and their primary current lamination in mixed sand–clay are significantly different from those in pure sand. Fractions of 1 to 10% of cohesive clay and <0·1% of EPS within a sand bed are sufficient to increase the development time of current ripples on a flat bed (Baas *et al*., 2013; Malarkey *et al*., 2015), and reduce the equilibrium height and wavelength of subaqueous dunes in mixed sand–clay (Schindler *et al*., 2015) and in mixed sand–clay–EPS (Parsons *et al*., 2016). This decrease in bedform size, as clay and EPS content are increased, is particularly pronounced for subaqueous dunes (Schindler *et al*., 2015; Parsons *et al*., 2016). However, it is hypothesised herein that current ripples in mixed sand–clay–EPS will also be smaller than in pure sand when developed within the time frame of a semidiurnal tide, because the additional time required to reach equilibrium dimensions compared to pure sand ranges from hours to tens of hours (Baas *et al*., 2013; Malarkey *et al*., 2015). A key process during bedform development in mixed sediment is the entrainment of clay, silt and EPS into suspension from a mixed sand–mud bed, i.e. winnowing (McCrone, 1962), because it may facilitate the bedforms eventually reaching heights and wavelengths that are similar to their mud‐free equivalents. This facilitative process appeared to be more important for current ripples (Baas *et al*., 2013; Malarkey *et al*., 2015) than for dunes (Schindler *et al*., 2015; Parsons *et al*., 2016). The above results were based exclusively on controlled laboratory experiments conducted with steady, uniform flow, constant flow depth, well‐sorted sand, a single type of clay mineral (kaolinite) and EPS (xanthan gum), and in fresh (Baas *et al*., 2013; Malarkey *et al*., 2015) or brackish (Schindler *et al*., 2015; Parsons *et al*., 2016) water.

The present paper compares, for the first time, the dynamics of experimental small‐scale bedforms in mixed sand–clay with those on an intertidal flat (Dee Estuary, north‐west England), based on integrated morphological and hydrodynamic data. Firstly, evidence is provided that current ripples found at low slack water at this field site and current ripples in laboratory experiments are influenced by physical and biological cohesion in a similar way. Thereafter, the inherently more complex field conditions are explored to elucidate the underlying processes that control the differences between the field data and the experimental data in the rate of change of current ripple wavelength with changing bed clay and EPS content. Finally, the wider implications of this validation study for predictive sediment transport models, bedform size predictors and sedimentary facies analysis are discussed.

## Methods

**Figure 1:**
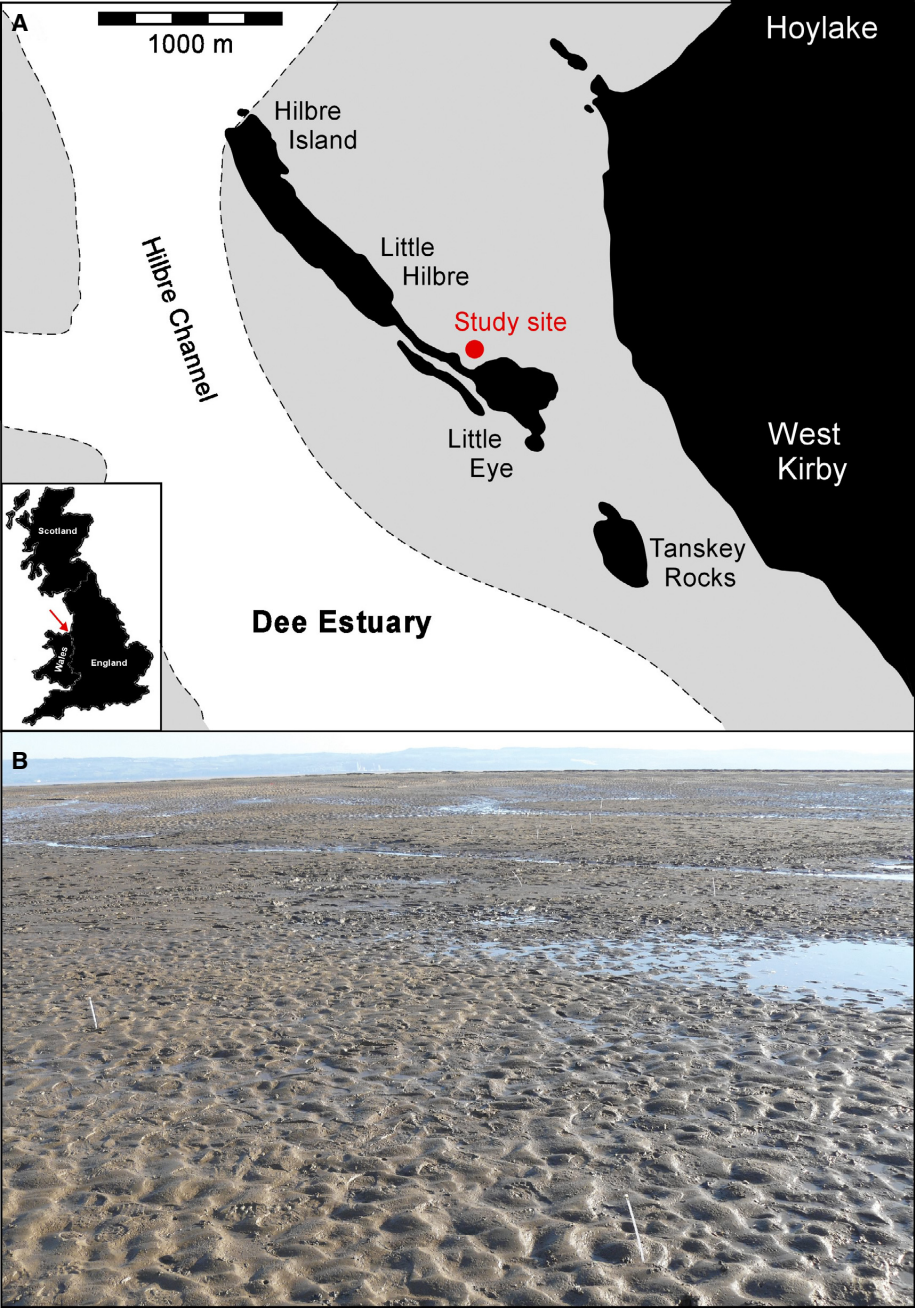
(A) Schematic map of the Dee Estuary around Hilbre Island, with the main tidal channel in white and the study area located on the grey‐coloured intertidal flat to the north‐west of Little Eye. The four islands are defined by the area above the mean high water mark and by any area of bedrock exposed at low water immediately below this mark. (B) Overview of study site. The ripples in the foreground have a wavelength of approximately 150 mm.

The data described herein integrate laboratory experiments with a comprehensive set of field measurements in an intertidal environment near Hilbre Island in the Dee Estuary, north‐west England (Fig. 1A). The Dee Estuary is funnel‐shaped and macrotidal, with a 7 to 8 m mean spring tidal range at Hilbre Island. Hilbre Island separates Hilbre Channel from intertidal flats west of the town of West Kirby (Fig. 1A). These tidal flats are flood‐dominated and rich in fine‐grained sediment (Moore *et al*., 2009). Waves are mainly generated locally within Liverpool Bay, with north‐westerly waves having the largest influence on sedimentary processes in the Dee Estuary (Brown & Wolf, 2009; Villaret *et al*., 2011).

The intertidal flats to the north‐west of Little Eye (Fig. 1A) have proven valuable as a natural laboratory for studying bedform dynamics in mixed sand–mud, owing to the large variety in sand–mud ratio, ranging from pure sand to sandy mud. During the field campaign in May 2013, the intertidal flats also contained significant amounts of EPS, ranging from 0·02% to 0·21% by weight (Lichtman *et al*., 2018). The present paper uses textural and morphological data collected from a 1400 m^2^ area on 26 May 2013, where conditions had been essentially wave‐free in the previous two tidal inundations. The bed was either flat, i.e. locally below movement threshold, or rippled with different sizes and planform patterns during exposure at low slack water (Fig. 1B). In order to study the relationship between these bedform properties and bed clay and EPS content, three plane beds and twelve rippled beds were described visually. Subsequently, using the method described in Appendix A, the three‐dimensional nature of the plan morphology of the ripples was characterised by the ripple planform index, *I*, where *I *>* *0·50 for 2D ripples, 0·39 ≤ *I *≤* *0·5 for 2D–3D ripples and *I *<* *0·39 for 3D ripples. The bedform wavelengths were measured by hand, because collecting bedform heights within the same low slack water period was not possible. Bulk sediment samples were collected between the crest and base of individual bedforms, and down to 20 mm below the base of these bedforms, for subsequent grain‐size analysis using a Malvern 2000 laser particle sizer (Malvern Panalytical Limited, Malvern, UK). This approach allowed comparison of surface sediment subject to winnowing (i.e. loss of fine particles and EPS to the water column) with deeper sediment unaffected by winnowing. The bed sampling also included flat, mud‐rich, surfaces without current ripples. For these flat beds, the subsurface samples were collected in the same way as for the rippled beds, i.e. down to 20 mm below the surface. These samples were supplemented by surface scrapes, several millimetres deep, to distinguish undisturbed sediment from sediment that may have been subjected to winnowing at the sediment–water interface. The sand fraction in the mixed sand–mud at the study site had a median grain diameter of 0·228 mm, and the mud fraction, encompassing all particles finer than 0·063 mm, ranged from 3·8 to 37·1% by volume. The mud fraction of the ripples at the field site contained 36% clay minerals by volume (standard deviation: 4%, *n *=* *7), based on X‐ray powder diffraction (XRD) data, obtained using the standard methodology for bulk sediment analysis (Moore & Reynolds, 1997). In decreasing order of abundance, the clay mineral assemblage comprised illite (68%), chlorite (16%), kaolinite (13%) and mixed layer illite/smectite (3%). This 36% is therefore inferred to represent the cohesive fraction within the mud fraction; the remaining 64% of the mud fraction was dominated by non‐cohesive quartz and feldspar. Following Lichtman *et al*. (2018), the cohesive clay fraction, *c*, can therefore be expressed in terms of the mud content, *m*, as:

(1) c=0·36m

thus yielding cohesive clay fractions ranging from 1·4 to 13·4% by volume. Because of time constraints during low slack water, it was not possible to collect EPS samples that exactly corresponded to these clay samples, since the former needed to be frozen in liquid nitrogen in the field (Hope, 2016). It was thus necessary to rely on the relationship between clay content and EPS content by weight, *e*, determined nearby (Lichtman *et al*., 2018):

(2) e=0·0105c+0·0302

(*R* ^2^ = 0·41, *P *<* *0·05, *n *=* *20), yielding EPS contents ranging from 0·04 to 0·17% by weight. Adjacent to the area where the bedforms were measured, near‐bed flow velocities were measured continuously during water cover using two frame‐mounted acoustic Doppler velocimeters (ADV) located at 0·25 m and 0·40 m above the sediment surface. The ADV data, with an acquisition rate of 0·2 Hz, was bin‐averaged over 30 s to determine Reynolds‐averaged velocities in the absence of waves. Immediately before the bedforms were studied on 26 May 2013, the field site experienced a spring tide with a maximum depth‐averaged flow velocity of 0·67 m s^−1^ (based on a total period of submergence of both probes of 4·7 h) and south‐eastward‐directed and north‐westward‐directed currents during flood and ebb, respectively. The maximum flow depth was 2·73 m close to high slack water. The depth‐averaged velocities were calculated from the vectorially added horizontal components of the ADV data and the flow depth, using the standard logarithmic law for wall‐bounded shear flow (Van Rijn, 1990).

## Relationship Between Ripple Properties and Bed Mud Content

### Results

**Figure 2:**
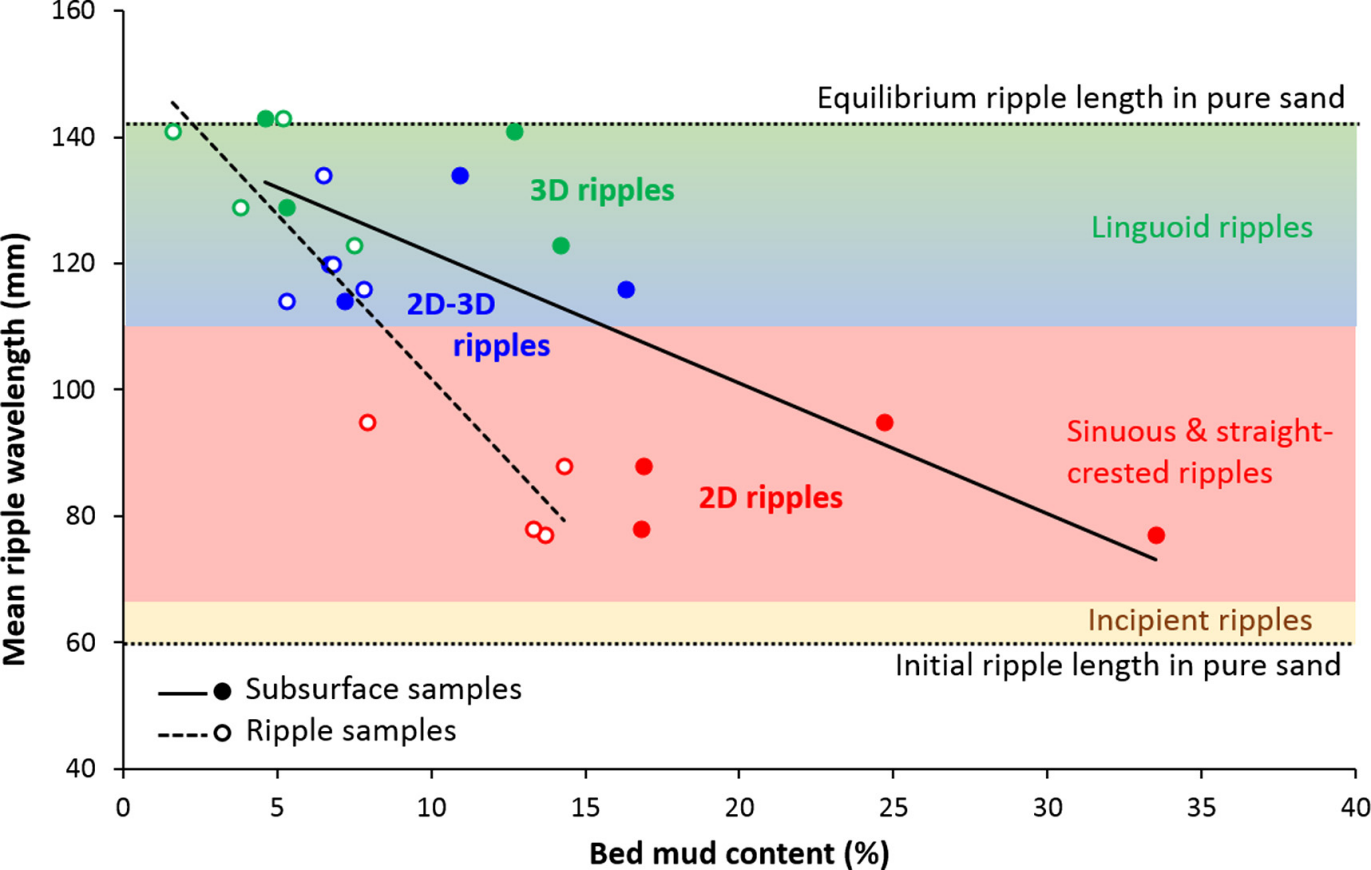
Mean ripple wavelength against bed mud content for the current ripples at the field site. The black continuous and dashed lines are the linear least‐square best fits for the subsurface and the ripple samples, respectively. The colours indicate incipient ripples (yellow), 2D sinuous and straight‐crested ripples (red), 2D–3D linguoid ripples (blue) and 3D linguoid ripples (green). The boundaries between these ripple types are from Baas (1999). The horizontal stippled lines denote the initial and equilibrium wavelengths of current ripples in 0·238 mm sand (*cf*. Baas, 1999).

**Figure 3:**
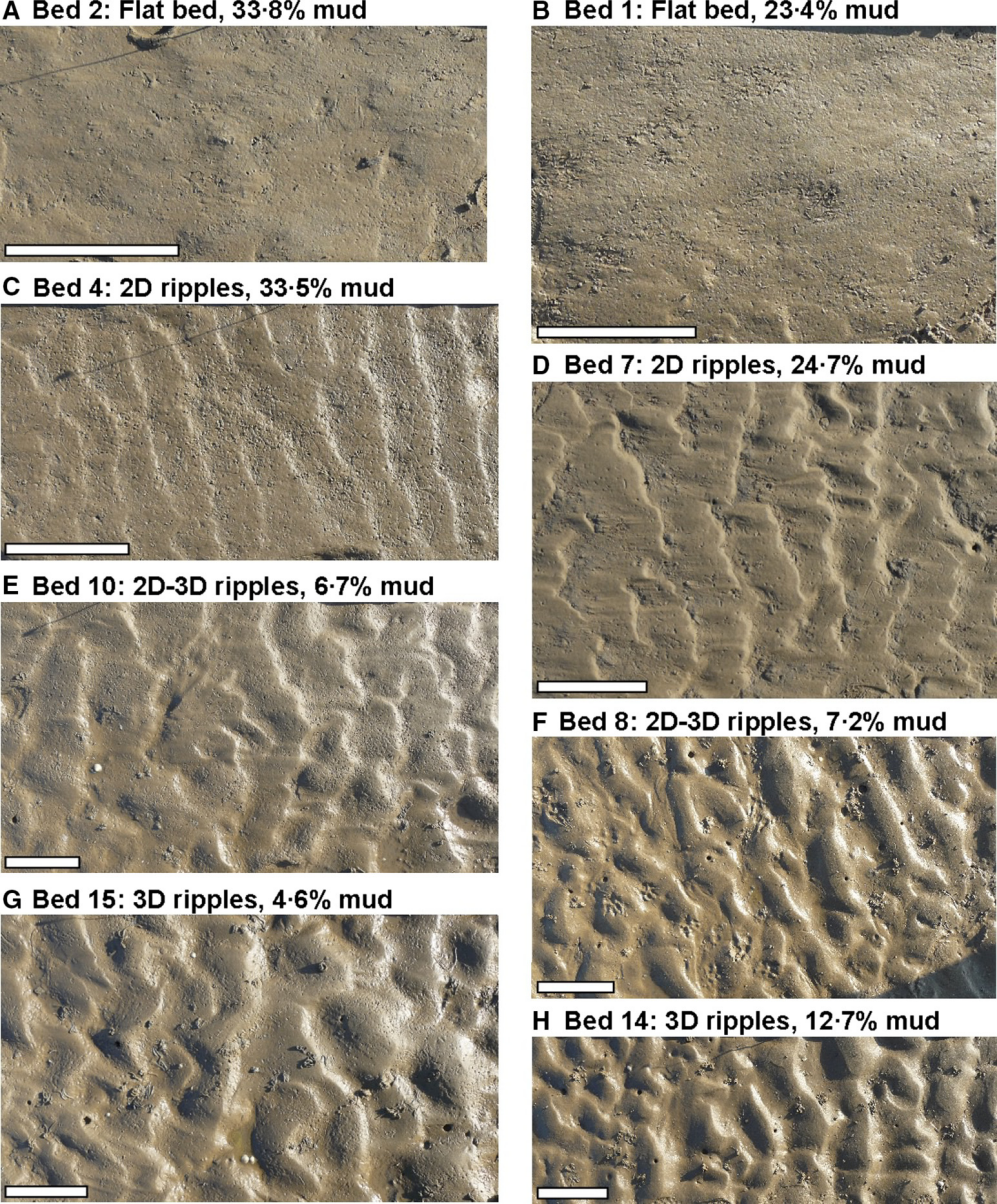
Field examples of flat beds (A) and (B), 2D ripples (C) and (D), 2D–3D ripples (E) and (F), and 3D ripples (G) and (H). Bed mud content decreases from (A) and (B) to (G) and (H). The percentages in (A) to (H) denote subsurface mud contents, as shown in Table 1. The scale bar is 200 mm long.

Table 1 provides the current ripple wavelength and planform index data, and the corresponding bed mud and cohesive clay measured at the study site. The mean ripple wavelength, *L*, is plotted against bed mud percentage within the ripples and in the subsurface underneath the ripples in Fig. 2. Figure 2 also provides information on the planform properties of these bedforms, and photographic imagery of the various plan morphologies is shown in Fig. 3.

**Table 1:** Summary of morphological and textural field data

Bed number	Ripple wavelength		Ripple planform index	Bed mud %		Bed clay %		Bedform type
	Mean (mm)	Number		Subsurface	Ripple^a^	Subsurface	Ripple^a^	
1	–	–	–	23·4	11·7	8·4	4·2	Flat bed
2	–	–	–	33·8	24·0	12·2	8·6	Flat bed
3	–	–	–	37·1	32·8	13·4	11·8	Flat bed
4	77	15	0·84	33·5	13·7	12·1	4·9	2D ripples
5	78	9	0·75	16·8	13·3	6·0	4·8	2D ripples
6	88	12	–	16·9	14·3	6·1	5·1	2D ripples
7	95	11	0·53	24·7	7·9	8·9	2·8	2D ripples
8	114	9	0·39	7·2	5·3	2·6	1·9	2D–3D ripples
9	116	8	0·41	16·3	7·8	5·9	2·8	2D–3D ripples
10	120	9	0·48	6·7	6·8	2·4	2·4	2D–3D ripples
11	134	7	0·41	10·9	6·5	3·9	2·3	2D–3D ripples
12	123	7	0·31	14·2	7·5	5·1	2·7	3D ripples
13	129	8	0·24	5·3	3·8	1·9	1·4	3D ripples
14	141	7	0·34	12·7	1·6	4·6	0·6	3D ripples
15	143	7	0·38	4·6	5·2	1·7	1·9	3D ripples

a Surface scrape for flat beds.

A statistically significant decreasing linear relationship between the mean ripple wavelength and the bed mud content, *m*, was found for the samples collected from the ripples and the subsurface (Fig. 2). These relationships are described by a linear trend with *R* ^2^ values of 0·44 (*P *=* *0·027 < 0·05, *n *=* *12) and 0·75 (*P *=* *0·00058 ≪ 0·05, *n *=* *12), respectively. The mud content in the subsurface was mostly greater than the mud content within the ripples, and this difference rapidly increased as the ripple wavelength decreased (Fig. 2). For the beds with the lowest mud content, the ripple wavelength was close to the equilibrium wavelength of current ripples in pure sand with a median grain size, *D*_50_, similar to the present study [*D*_50_ = 0·238 mm and *L *=* *141 mm (Baas, 1999) versus *D*_50_ = 0·228 mm and *L *=* *142 mm herein]. The smallest ripples, formed at the highest bed mud contents, were approximately 20 mm longer than incipient current ripples in the experimental study of Baas (1999) (Fig. 2).

Figure 3A and B shows examples of flat surfaces without ripples, which were found in concert with high subsurface mud contents of 23·4%, 33·8% and 37·1% (Table 1). As observed for the rippled beds, the mud content in the surface scrapes of the flat beds was consistently lower than in the subsurface samples down to 20 mm (Table 1). The average mud content in this surficial layer was 23%, which was higher than any of the mud contents within the ripples. The current ripples with *L *≤* *95 mm (Table 1) had straight to weakly sinuous crest lines (Fig. 3C and D); these bedforms are referred to as two‐dimensional (2D) ripples in the remainder of this paper (Fig. 2). The ripples with a wavelength >110 mm were either three‐dimensional (3D) ripples with linguoid crests (Fig. 3G and H) or transitional between the 2D and 3D ripples (Fig. 3E and F). The beds covered in 2D–3D ripples consisted of patches of linguoid ripples next to ripples with more continuous crests. With one exception, the 2D–3D ripples were shorter in wavelength than the 3D ripples (Fig. 2).

### Interpretation

These field data reveal a decreasing linear relationship between bed mud content and ripple wavelength, suggesting that clay minerals within the bed hindered the near‐bed movement of non‐cohesive grains (Mitchener & Torfs, 1996; Baas *et al*., 2013; Wang & Sturm, 2016). Consequently, the tidal currents were able to form 3D linguoid current ripples only in beds with *m *<* *11% in the subsurface and *m *<* *6% within the ripples. At progressively higher bed mud contents, cohesive forces restricted bedform development to smaller 2D–3D and 2D current ripples. The boundary between these ripple types was at bed mud contents in the subsurface and within the ripples of 18% and 10%, respectively. The beds were too cohesive for any ripple development at subsurface bed mud contents of 31 ± 7% and surficial mud contents of 23 ± 11%, where 7% and 11% denote standard deviations of the mean.

The wavelength and planform morphology of the field ripples correspond remarkably well with the laboratory ripples in 0·238 mm sand of Baas (1999). The field ripples were too large to be classified as the incipient ripples of Baas (1999), which were up to 67 mm long. The wavelength of the 2D field ripples was well within the limits of the straight‐crested and sinuous ripples of Baas (1999) (Fig. 2), and the 2D–3D ripples and most 3D ripples at the field site correspond in wavelength to the non‐equilibrium linguoid ripples of Baas (1999). Only at the lowest bed mud contents did the 3D ripples reach wavelengths that were similar to the equilibrium linguoid ripples in the clay‐free sand of Baas (1999).

## Comparison with Experimental Mixed Sand–Clay Ripples

**Figure 4:**
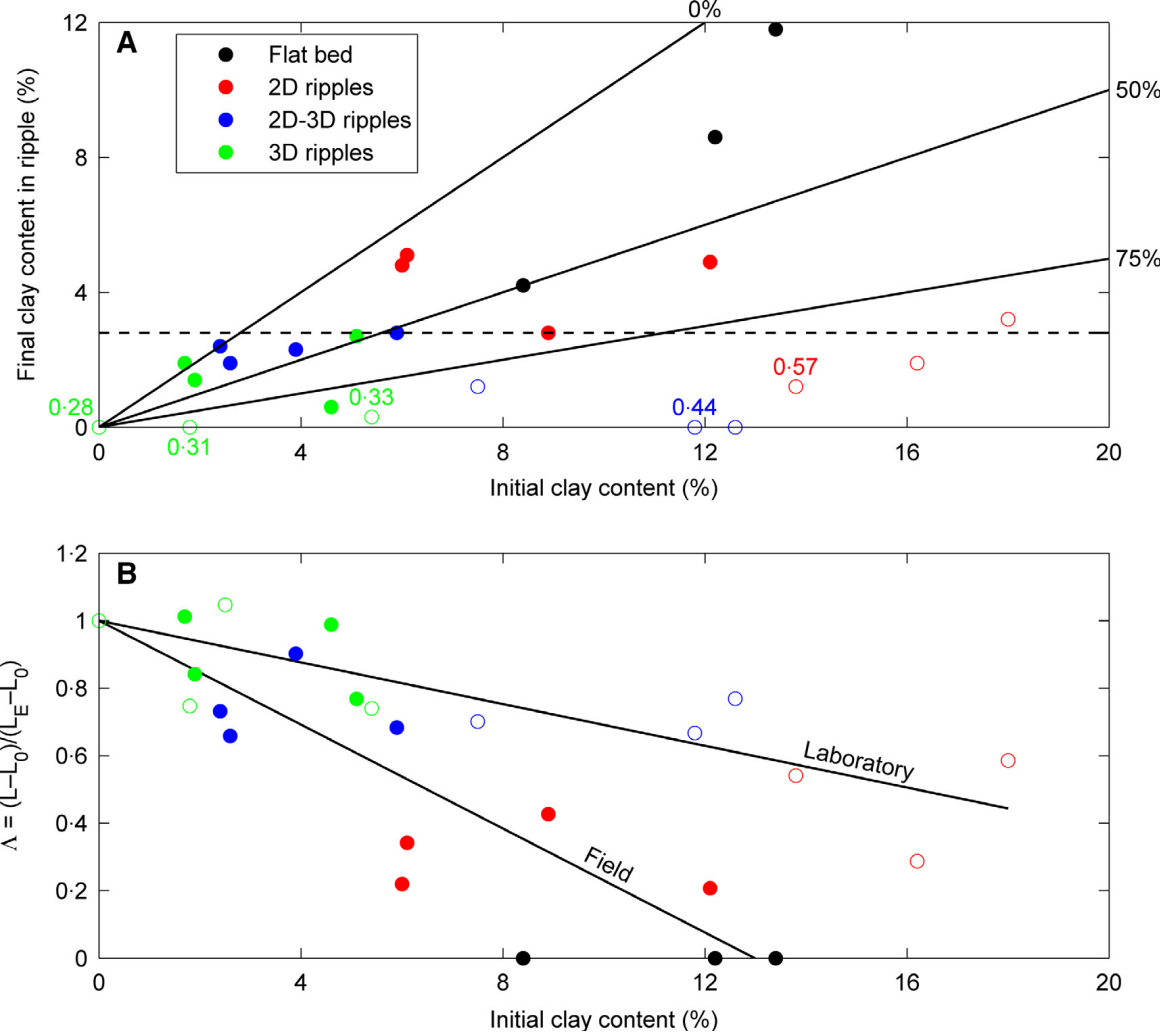
Final clay content (A) and dimensionless current ripple wavelength (B) against initial bed clay content for the ripples at the field site, with *D*_50_ = 0·228 mm, based on the subsurface samples (solid circles) and in the experiments of Baas *et al*. (2013) with *D*_50_ = 0·143 mm (open circles). In (A), the black continuous lines represent different winnowing efficiencies, the dashed line corresponds to 2·8% final clay content, which is the upper limit of occurrence for 2D–3D and 3D ripples for the field data and the laboratory data, and the coloured numbers are ripple planform indices (Appendix A), for the closest experimental case for which plan‐view images were available. In (B), the black continuous and dashed lines are the linear least‐square best fits for the field data and the experimental data, respectively. The colours indicate the bed configurations, as in (A).

To facilitate a direct comparison of the ripples at the field site with the ripples in the mixed sand–clay experiments of Baas *et al*. (2013), the bed mud contents in the subsurface samples collected at the field site, shown in Fig. 2, were converted to cohesive clay contents using Eq. (1). The difference in cohesive clay content in the samples from the subsurface and the ripples agrees with previous studies that associated the development of bedforms with the winnowing of fine particles (Baas *et al*., 2013; Schindler *et al*., 2015; Parsons *et al*., 2016; Lichtman *et al*., 2018). The winnowing depth is limited by the height of the bedforms, which explains why the clay content in the subsurface is up to three times higher than the clay content in the ripples on the surface (Table 1). However, as shown in Fig. 4A, the winnowing of fine sediment in the field was not as efficient as in the laboratory experiments. Defining the boundaries of winnowing efficiency as 100% for full removal of bed clay and as 0% for full retention of clay during bedform development, the average winnowing efficiency at the field site was 35%, compared to 93% in the experiments of Baas *et al*. (2013). This low winnowing efficiency may be associated with physical and biological processes that counteract winnowing and add and store clay in the bed under field conditions, such as advection of suspended clay particles, deposition of mud flocs during high slack tide, mixing of clay and silt into the bed by benthic organisms, and hyporheic pumping (Packman *et al*., 2000; Blois *et al*., 2014). Following the approach of Malarkey *et al*. (2015), described in Appendix B, the time timescale of hyporheic pumping into the bed – a minimum of 19·7 h equivalent to four inundation periods – is far slower than the winnowing associated with ripple overturning (0·2 to 0·4 h). Winnowing appears to have taken place also on the strongly cohesive flat beds, although this process might have been limited to the uppermost millimetres, and the reduction of bed mud content was insufficient to enable ripple formation within the time available. In addition, the production, accumulation, breakdown and loss of EPS is complex given its multiple sources (bacteria, microphytobenthos and infauna; Decho & Gutierrez, 2017) compared to the rather simple situation represented in the laboratory with known initial concentrations of EPS, no active production and xanthan gum being used as a proxy for EPS.

In order to futher facilitate comparisons between the field and laboratory data, the ripple wavelengths were non‐dimensionalised to exclude the influence of grain size on current ripple dimensions (Raudkivi, 1997; Baas, 1999; Soulsby & Whitehouse, 2005):

(3) $$\Lambda =\frac{L-L_{0}}{L_{E}-L_{0}}$$

where Λ is the dimensionless ripple wavelength and *L*_*E*_ and *L*_0_ are the wavelengths of equilibrium ripples and incipient ripples, first appearing on a flat bed, in pure sand. Since Λ = 0 for *L = L*_0_ and Λ = 1 for *L = L*_*E*_, Λ can also be thought of as the relative age of the ripple. *L*_*E*_ and *L*_0_ are functions of grain size. Herein, *L*_*E*_ = 142 mm and *L*_0_ = 60 mm for the 0·228 mm sand at the field site, where *L*_0_ is taken from the experimental data for 0·238 mm sand of Baas (1999), and *L*_*E*_ = 115·9 mm and *L*_0_ = 28·5 mm for the 0·143 mm sand used in the experiments of Baas *et al*. (2013). The experiments of Baas *et al*. (2013) were conducted with steady, uniform, freshwater flow at a depth‐averaged velocity of 0·36 m s^−1^ and the current ripples were given 2 h to develop on a flat bed consisting of mixed sand and kaolin clay.

Figure 4B demonstrates that the dimensionless wavelength of the current ripples decreased linearly with increasing initial cohesive clay content in the bed for both the field data and the laboratory data. Moreover, both datasets show a change from 3D ripples via 2D–3D ripples to 2D ripples, as the initial cohesive clay content in the bed was increased (Baas *et al*., 2013), and these planforms are demarcated by similar Λ values and *I* values (Fig. 4B; Table 1). The flat‐bed data points from the field site, which were excluded from Fig. 2, can now be included, because they correspond to Λ = 0. The laboratory experiments thus provide an appropriate analogue for the effect of cohesive forces on current ripple size and planform morphology under estuarine conditions. However, the rate of decrease in ripple wavelength was significantly greater for the field ripples than for the experimental ripples. The field data also show more scatter than the laboratory data, particularly for high clay contents. Linear extrapolation predicts a total absence of ripple development at 13% cohesive clay at the field site (Fig. 4B).

This difference in the rate of change in ripple wavelength may be caused by physical and biological variables that were omitted from the flume experiments of Baas *et al*. (2013). Below, the possible influence on bedform dynamics of the following main variables is assessed: (i) clay mineral type; (ii) current shear stress; (iii) bed EPS content; and (iv) pore water salinity. Increased current shear stresses are expected to promote the development of current ripples on the intertidal flat, whereas strongly cohesive clay minerals, the presence of biological cohesion in the form of EPS, and seawater salinity, which may modify biological and phyiscal cohesion, should hinder the development of the current ripples. Each of these variables is discussed in detail below and, if possible, its relative contribution to the difference in the rate of change of ripple wavelength is determined (Fig. 4B).

## Discussion of Specific Controls on Ripple Dynamics at the Field Site

### Clay type

Kaolinite clay was used by Baas *et al*. (2013) in laboratory experiments, whereas illite clay was dominant at the field site. Illite is more cohesive than kaolinite because illite particles have a larger specific surface area and a larger cation exchange capacity than kaolinite particles (Hillel, 2004; Yong *et al*., 2012). Mixtures of sand and illite should, therefore, have a higher yield strength than mixtures of sand and kaolinite, and bedforms might develop more readily in mixed sand–kaolinite. However, Baker *et al*. (2017) showed that the difference in yield strength between kaolinite and montmorillonite (including bentonite) is small for volumetric clay concentrations below 10%. This result agrees with an experimental study by Torfs (1995, in Van Ledden, 2001), in which the critical clay content for cohesive behaviour was 3 to 4% for both kaolinite and montmorillonite. Since illite is less cohesive than montmorillonite, all of the current ripples at the field site were formed at bed clay fractions below 13%, and the subsidiary clay minerals kaolinite and chlorite in the field samples have lower yield strength than the illite, it is inferred that the effect of clay type on the difference in the rate of change of ripple wavelength between the field data and the Baas *et al*. (2013) laboratory data was small.

### Current shear stresses

**Figure 5:**
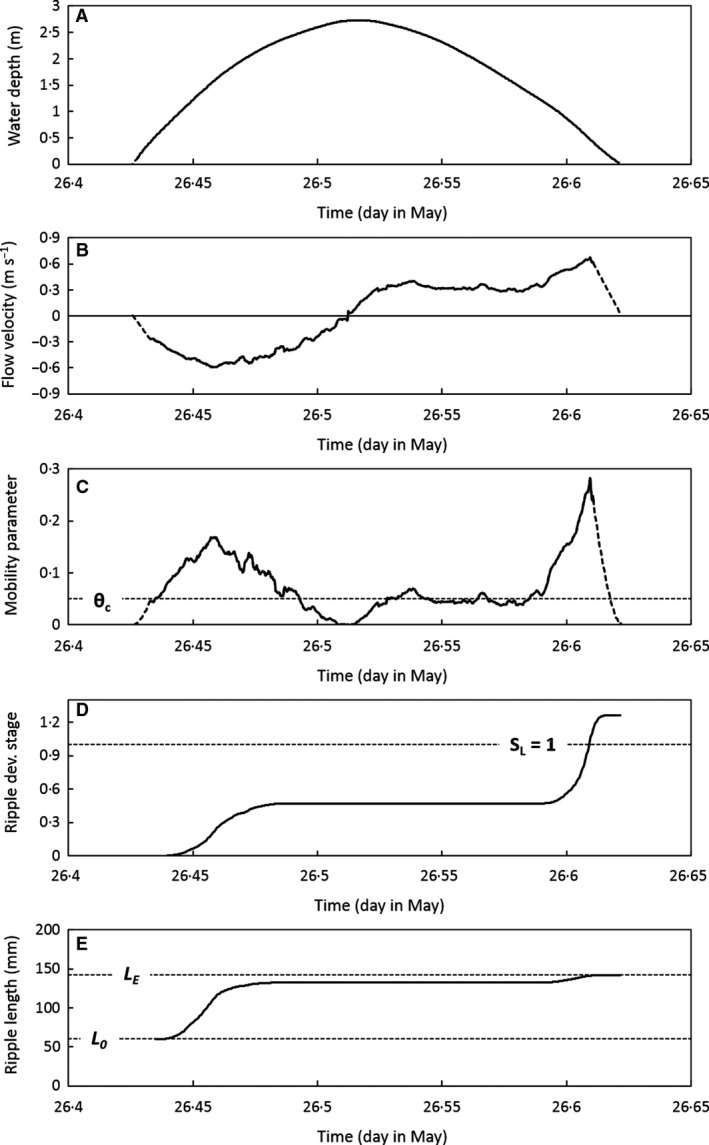
Summary of the field data, collected on 26 May 2013: (A) water depth; (B) depth‐averaged flow velocity; (C) grain‐related mobility parameter; (D) cumulative ripple wavelength development stage; (E) predicted temporal development of ripple wavelength. The dashed lines in (B) and (C) denote linear extrapolations. *θ*_*c*_ = 0·0508 is critical grain‐related mobility parameter; *L*_*E*_ = 142 mm is equilibrium ripple wavelength; *L*_0_ = 60 mm is initial ripple wavelength. *S*_*L*_ is cumulative ripple wavelength development stage.

The experiments of Baas *et al*. (2013) were conducted at a constant depth‐averaged flow velocity of 0·36 m s^−1^, whereas the field site was subjected to unsteady flow with a maximum depth‐averaged flow velocity of 0·67 m s^−1^ (Fig. 5B). As the rate of bedform development increases non‐linearly with flow velocity (Baas, 1994, 1999), the current ripples at the field site could have been preserved at the end of ebb flow in a different development stage than the mixed sand–clay ripples of Baas *et al*. (2013). This development stage would render these ripples *non‐equilibrium* bedforms, *sensu* Baas (1994). Before testing this hypothesis, it is important to consider the alternative that the ripples on the intertidal flat were smaller than ripples in the equivalent clay‐free sand because they are *equilibrium* bedforms (Baas, 1994) with wavelengths that decrease with increasing bed clay content. Baas *et al*. (2013) discussed both possibilities without stating a preference, but, subsequently, Malarkey *et al*. (2015) provided experimental evidence that current ripples in cohesive mixtures of sand and EPS reach equilibrium heights and wavelengths that are similar to those in non‐cohesive sand, provided that the flow duration is sufficiently long. Since the Baas *et al*. (2013) ripples were essentially free of clay (Fig. 4A), and probably still developing at the end of their experiments, it can be assumed that this evidence can be extrapolated from biological cohesion to physical cohesion. Thus the mathematical model of Oost & Baas (1994) and Baas *et al*. (2000) for current ripple development in unsteady flow was used to compare the development stage of the ripples at the field site and in the experiments of Baas *et al*. (2013):

(4) $$\frac{L_{\mathrm{SL}}-L_{0}}{L_{E}-L_{0}}=1-0\cdot 01^{S_{L}}\wedge S_{L}=\int_{0}^{T}\frac{\mathrm{dt}}{T_{E}\left(t\right)}$$

(5) $$T_{E}={\left(\frac{\theta^{\prime }-\theta_{c}}{a}\right)}^{-\frac{1}{b}}$$

In Eq. (4), *L*_*SL*_ is the ripple wavelength at *T*,* T* is the flow duration in hours, *S*_*L*_ is the cumulative development stage of ripple wavelength and *T*_*E*_(*t*) is the equilibrium time in hours as a function of time *t*, i.e. the time needed to reach equilibrium ripple wavelength at the acting current strength: *S*_*L*_ = 0 for a flat bed, and *S*_*L*_ ≥ 1 for equilibrium ripples. In Eq. (5), *T*_*E*_ is related to the inverse of the flow forcing:

(6) $$\theta^{\prime }=\frac{U^{2}}{\left(\rho_{s}/\rho -1\right)C^{\prime 2}D_{50}}$$

(7) $$C^{\;\prime }=18\log\left(\frac{12h}{3D_{90}}\right)$$

where *θ′* is the grain‐related mobility parameter, *U* is the depth‐averaged flow velocity, *ρ*_*s*_ = 2650 kg m^−3^ is the sediment density, *ρ = 1*027 kg m^−3^ is the density of seawater, *D*_50_ is the median grain size of the bed sand fraction, *C′* is the grain‐related Chézy coefficient, *h* is the flow depth and *D*_90_ is the 90th percentile of the grain‐size distribution of the sand fraction (herein, *D*_90_ = 0·364 mm).

In Eq. (5), *θ*_*c*_ represents the critical mobility parameter for particle entrainment, parameterised from the Shields curve (Shields, 1936) by Soulsby (1997):

(8) $$\theta_{c}=\frac{0\cdot 3}{1+1\cdot 2D_{*}}+0\cdot 055\left(1-e^{-0\cdot 02D_{*}}\right)$$

(9) $$D_{*}={\left(\frac{(\rho_{s}/\rho -1)g}{\nu^{2}}\right)}^{1/3}D_{50}$$

where D_*_ is the dimensionless grain size parameter, *g *=* *9·81 m s^−2^ is the acceleration due to gravity and *ν = 1*·37 × 10^−6^ m^2^_ _s^−1^ is the kinematic viscosity of seawater at 10°C. The coefficients *a* and *b* in Eq. (5) are grain‐size dependent. For the field site with 0·228 mm sand herein, *a *=* *0·112 and *b *=* *0·473, which are linearly interpolated from values for 0·095 mm and 0·238 mm sand (*cf*. Baas *et al*., 2000).

Equations (4), (5), (6), (7), (8), (9) to (4), (5), (6), (7), (8), (9) were used to predict the final wavelength of clay‐free current ripples developing on a flat bed during the flood and ebb tides immediately preceding the data collection (Fig. 5). The grain‐related mobility parameter (Fig. 5C) was calculated from the water depth (Fig. 5A) and the depth‐averaged flow velocity (Fig. 5B). In turn, *θ′* was used to predict the cumulative ripple development stage (Fig. 5D) and finally the temporal development of ripple wavelength (Fig. 5E). As expected, the highest rates of ripple wavelength development occurred around maximum flood and maximum ebb flow. The ripples were predicted to attain their full equilibrium wavelength after 4·4 h of the available 4·7 h of tidal flow (Fig. 5D and E). The model predicts *S*_*L*_ = 1·263 at time *T*, with *L*_*SL*_ = 141·8 mm. However, the age of the sandiest ripples at the field site might extend further back in time, because it is unknown how long these bedforms had been at equilibrium. Yet, it is unlikely that these bedforms formed more than three or four principal lunar semi‐diurnal tidal cycles before the ripple wavelength data were collected because strong waves on 24 May 2013 generated wave ripples, combined flow ripples, and upper‐stage plane beds (Lichtman *et al*., 2018). The model predictions for *S*_*L*_ and *L*_*SL*_ in Fig. 5D and E should, therefore, be considered minimum values.

**Figure 6:**
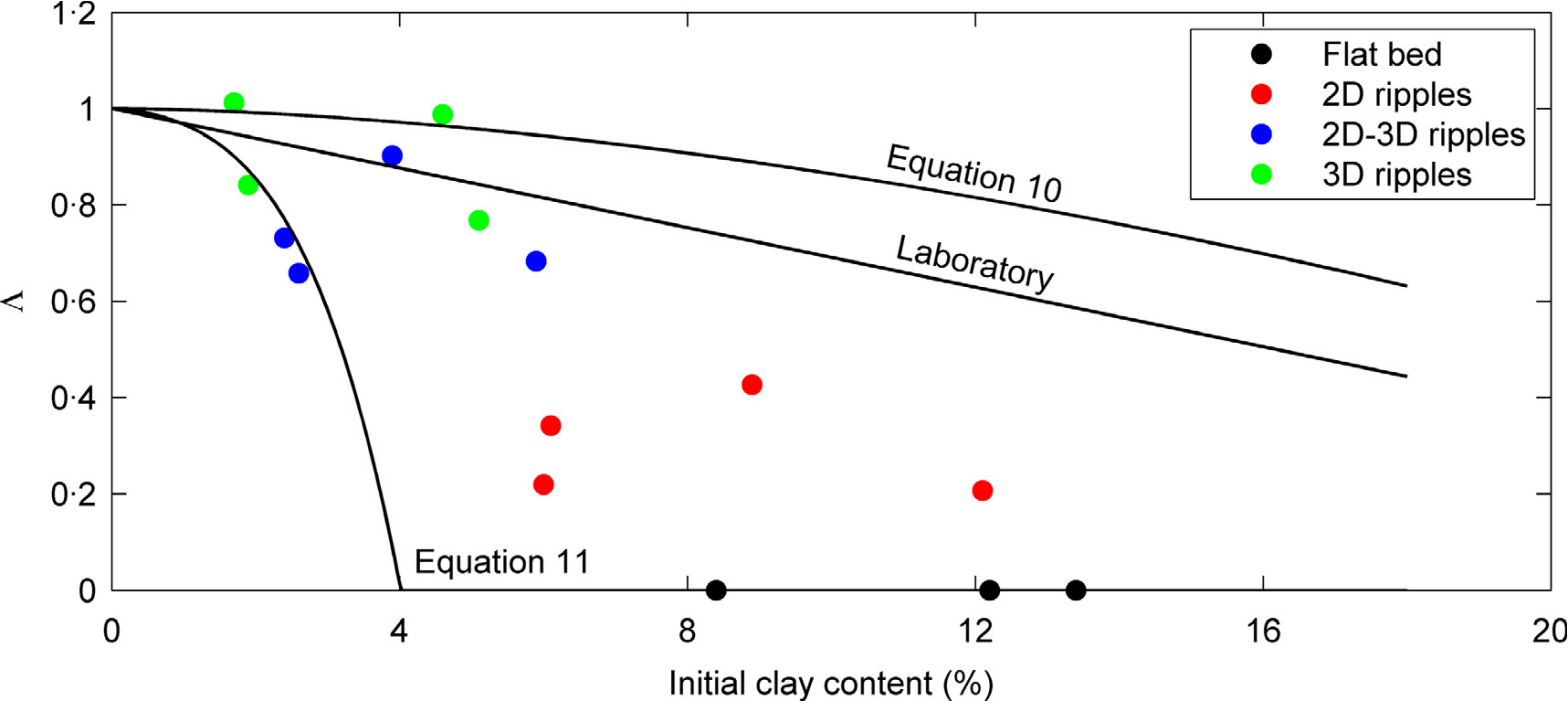
Dimensionless current ripple wavelength against initial bed clay content for the ripples in the field. All lines relate to the experimental data. ‘Laboratory’ is the least‐square best fit from Fig. 4B; ‘Equation (10)’ is the adjustment of the ripple development stage to that of the field data, *S*_*L*_ = 1·26. ‘Equation (11)’ also includes the initiation time for ripples, based on the mixed sand‐EPS experiments of Malarkey *et al*. (2015). The symbols and their colours represent the field data, as in Fig. 4.

In the experiments of Baas *et al*. (2013), the equilibrium wavelength of the current ripples in pure sand, based on *L*_*SL*_ = 0·99 *L*_*E*_ (*cf*. Eq. (4)), was reached at 2·7 h. Since the experiments with mixed sand–clay were halted after 2 h, this equilibrium time corresponds to *S*_*L*_ = 2/2·7 = 0·74, which can be compared with the *S*_*L*_ value in the field of 1·26 by adjusting the laboratory data of Baas *et al*. (2013) according to:

(10) $$\Lambda =1-{\left(\frac{L_{E}-L_{0\cdot 74}}{L_{E}-L_{0}}\right)}^{\frac{1\cdot 26}{0\cdot 74}}$$

where *L*_0·74_ is the ripple wavelength after 2 h. Equation (10) is represented by the upper curve in Fig. 6. This curve is above the curve for the laboratory data (Fig. 6) because the experiments have been adjusted as though they were run over a longer period: 1·26 × 2·7 = 3·4 h. Forcing the relative ages of the laboratory and field data to be the same in this way has resulted in an increase in the difference between these datasets. Hence, current shear stress cannot explain the observed difference in the rate of change of ripple wavelength between the field data and the laboratory data.

### Extracellular polymeric substances content in the bed

An important difference between the field data and the laboratory data of Baas *et al*. (2013) is the lack of biological cohesion in the flume experiments. The EPS content in the bed samples from the field site was not measured directly, as explained earlier, but Eq. (2) can be used to make a qualitative assessment of the effect of EPS on the remaining difference in the rate of change of dimensionless ripple wavelength with increasing bed clay content (Fig. 6). The subsurface samples contained up to 0·194% EPS (Eq. (2)), which is comparable to the bed EPS content in the laboratory data of Malarkey *et al*. (2015). According to Malarkey *et al*. (2015), the time needed for incipient ripples to appear on the flat bed, *t*_*i*_, is 0·1, 0·4, 1·0, 2·7 and 7·9 h for values of *e* of 0%, 0·015625%, 0·03125%, 0·0625% and 0·125%, respectively. This can be expressed as *t*_*i*_ = 0·1 + 204·1*e* ^1·57^, where *e* is given by Eq. (2), such that an effective *S*_*L*_ can be defined as (3·4–*t*_*i*_)/2·7 = 1·26–0·37*t*_*i*_, where *S*_*L*_ is forced to be greater than 0, and gives Λ as:

(11) $$\Lambda =1-{\left(\frac{L_{E}-L_{0\cdot 74}}{L_{E}-L_{0}}\right)}^{\frac{1\cdot 26-0\cdot 37t_{i}}{0\cdot 74}}$$

which is the curve labelled ‘Equation (11)’ in Fig. 6. This curve represents a conservative estimate of the effect of EPS, since Eq. (11) does not include the considerably longer time needed to develop from incipient to equilibrium linguoid current ripples in mixed sand–EPS as opposed to pure sand (Malarkey *et al*., 2015). Despite being a conservative estimate, the calculated effect of xanthan gum on ripple wavelength development rate is still considerably stronger than the observed effect (Fig. 6). Yet, Eq. (11) captures the three flat‐bed cases reasonably well. It is likely that the effect of xanthan gum on bedform development is stronger than that of naturally occurring EPS (Tolhurst *et al*., 2002; Van de Lageweg *et al*., 2017). Xanthan gum is strongly cohesive, and has a constant distribution in the vertical, so the experiments may not be representative of estuarine conditions, where EPS typically reaches a maximum concentration in biofilms at the sediment surface (Taylor & Paterson, 1998). The present authors thus conclude that the difference between the field and laboratory results can reasonably be explained by the presence of EPS in the bed samples from the Dee Estuary.

**Figure 7:**
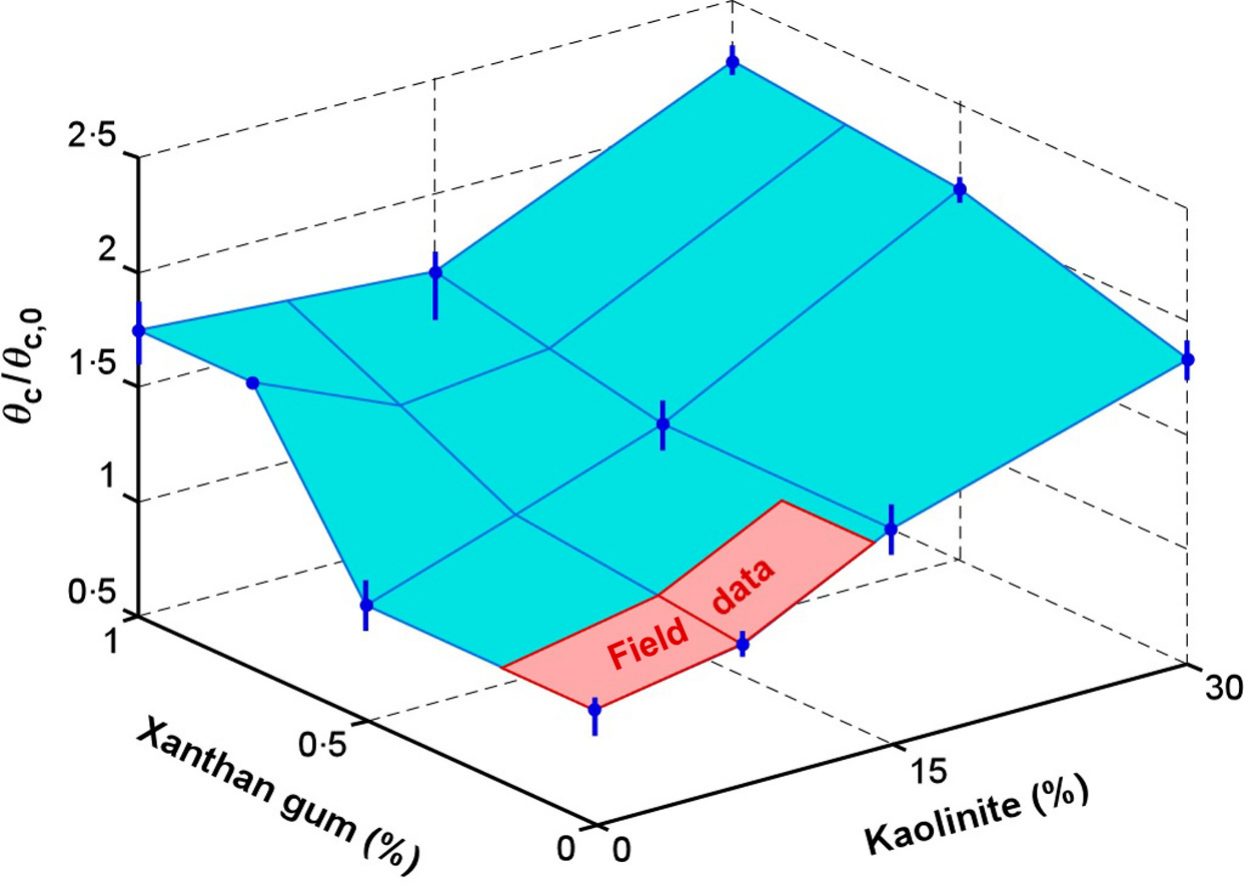
Relative change in critical Shields parameter (including standard error) for sand with kaolin clay, sand with EPS‐proxy xanthan gum, and sand with mixtures of kaolin and xanthan gum. The term *θ*_*c,*0_ on the vertical axis denotes the critical Shields parameter for pure sand with a median grain diameter of 0·148 mm. The blue coloured surface denotes the laboratory data. The red coloured insert denotes the approximate range of the field data.

The long delay in the initiation, as well as the long development time, of the current ripples in the EPS–sand experiments (Malarkey *et al*., 2015), are likely to be related to increases in the threshold of motion. Thus, the flows need to first lower the threshold of motion through winnowing of EPS before ripples begin to form. Based on a separate series of flume experiments (see Appendix C for further details), Fig. 7 provides an estimation of the increase of *θ*_*c*_ for kaolin clay and the xanthan gum, in which the critical Shields parameter was determined for mixed sand–clay–EPS by gradually increasing the bed shear stress imposed by a steady, uniform flow until a downstream bedload trap started to collect sediment particles. For up to 1% xanthan gum and up to 30% kaolin, *θ*_*c*_ was up to 73% and 83% higher than for the non‐cohesive reference sand. The combined effect of biological and physical cohesion led to an increase in *θ*_*c*_ by up to 123%. For the field site, where the current ripples formed only below 13% clay and below 0·2% EPS, Fig. 7 suggests a maximum increase in *θ*_*c*_ by 27% for the mixed sand–clay–EPS. It should be emphasised, however, that this percentage increase is merely an approximation of the combined effect of physical and biological cohesion on *θ*_*c*_ because the experiments were conducted with types of sand, clay and EPS that were different from those at the field site.

### Water salinity

The laboratory experiments of Baas *et al*. (2013) were conducted with freshwater, whereas the current ripples at the field site were formed in seawater. Salinity is well‐known to promote the formation of cohesive bonds between clay particles through the availability of cations that neutralise the negatively charged edges of clay platelets and therewith stimulate the attraction of these platelets by van der Waals forces (Manning *et al*., 2007; Mehta, 2013). This physicochemical attraction leads to the development of clay aggregates, i.e. floccules, that often have higher settling velocities than their constituent particles. The effect of salinity is larger for suspended sediments than for erosion thresholds. Experimental studies typically find a positive correlation between erosion threshold and salinity for muddy sediment (e.g. Kandiah, 1974; Parchure & Mehta, 1985), but this is not always the case. For instance, no significant statistical relationship between salinity and critical shear stress existed for mixed sand—EPS in the laboratory experiments of Van de Lageweg *et al*. (2017). Furthermore, the relationship between salinity and critical shear stress for sediment motion in field studies is ambiguous. Amos *et al*. (2003) found no difference in the threshold of erosion for natural muds in marine and freshwater environments, whereas Debnath *et al*. (2007) obtained a negative correlation between salinity and erosion rate for natural muds, and Spears *et al*. (2008) found that the erosion threshold increased seaward with salinity in the Eden Estuary (south‐eastern Scotland). These differences between the field studies, in comparison to theoretical and experimental approaches, suggest that at least in some cases the effect of salinity on bed erosion is masked by other parameters in the natural environment, such as bed density and EPS content (e.g. Grabowski *et al*., 2011). For the present study, it is postulated that the difference in the rate of decrease of dimensionless ripple wavelength, as cohesive clay content is increased, between the field data and the experimental data (Fig. 6) is partly caused by the difference in pore water salinity. However, based on the available literature, this effect is expected to be secondary. Some support for salinity having a secondary effect is the consistency in the development of ripples in freshwater (Baas *et al*., 2013; Malarkey *et al*., 2015), and ripples and dunes in brackish water (Schindler *et al*., 2015; Parsons *et al*., 2016).

## Wider Implications

### Frequency of occurrence of equilibrium and non‐equilibrium current ripples

At the field site, the presence of cohesive clay and EPS was found to have had the greatest influence on the growth rate and size of the current ripples, followed in decreasing order of influence by flow forcing and salinity, whereas the influence of clay type was small to negligible. However, the relative contribution of these controlling variables is likely to be unique to the measurement period at the study site.

Physical and biological cohesion are expected to have a strong influence on current ripple dynamics because small fractions of clay and EPS, akin to most aqueous environments, are sufficient to reduce current ripple size, promote two‐dimensionality of ripples and delay ripple growth. In environments where the currents and waves are stronger than at the field site, for example in tidal channels and on wave‐facing beaches, sand‐rich equilibrium bedforms are probably more common, because stronger hydrodynamic forcing promotes the development rate of the bedforms, assisted by a faster rate of winnowing of clay and EPS from the bed. This raises the question of whether non‐equilibrium mixed sand–clay–EPS ripples will dominate the bed when the currents are weaker than at the field site. In fact, this weaker hydrodynamic forcing should be the more likely scenario because the mean spring tidal range of 7 to 8 m in the Dee Estuary is amongst the largest in Europe, and the field data were collected at spring tide in this macrotidal estuary. Under such circumstances, maximum tidal velocities are higher than in many microtidal, mesotidal and other macrotidal environments. However, since the formation of the current ripples in this study was restricted to bed clay and EPS fractions of up to only 13·0% and 0·2%, it is inferred here that mixed sand–clay–EPS ripples form less readily on sand–mud tidal flats in these lower‐energy environments, even under the consideration that these percentages might vary with clay type, EPS type and flow forcing. Assuming that it is possible to form bedforms at all in such conditions, many tidal cycles would be needed to form recognisable mixed sand–clay–EPS ripples, and their preservation would depend on the frequency of occurrence of processes that destroy these bedforms, such as bioturbation and storm waves. Mixed sand–clay–EPS ripples might be restricted to a narrow strip at the boundary between sandy tidal channels and muddy tidal flats in estuaries with a smaller tidal range than the study site. Likewise, it is anticipated that during neap tides in the Dee Estuary, and in other macrotidal estuaries with a similar tidal range, the mixed sand–clay–EPS bedforms shift from intertidal flats towards areas of confined tidal flow, in particular subtidal channels.

Although the effect of pore water salinity on current ripple growth rate and size is not fully understood at present, the concentration of cations in brackish water and seawater is expected to be large enough to cause longer delays in the development of mixed sand–clay–EPS current ripples in fully marine and estuarine environments than in freshwater environments, such as rivers and lakes. It is therefore hypothesised for the benefit of future studies that equilibrium mixed sand–clay–EPS bedforms are more common in freshwater. At the field site, the influence of clay type was found to be small. However, tidal flows with significantly larger velocities than at the field site, and possibly helped by surface water waves, might be able to form current ripples at *c *>* *13% and *e *>* *0·2%, for which differences in yield strength between clay types start to play a more significant role in causing differences in bed cohesion (Baker *et al*., 2017), and therefore bedform development rate.

### Implications for studies in modern environments

Accurate knowledge of the geotechnical, morphological and biological properties of the seabed is essential for the quantification of near‐bed sediment transport in modern environments. These properties include the sediment size, the cohesive strength of the bed, the critical shear stress for sediment motion, and the size and migration rate of bedforms. The rate of bedload transport per unit width, *q*_*b*_, can be calculated based on the volumetric sediment concentration, *C*_*b*_, the particle velocity, *u*_*p*_, and the saltation height of the particles, *δ*_*b*_ (Van Rijn, 1993):

(12) $$q_{b}=C_{b}u_{p}\delta_{b}$$

or on the current ripple height, *H*, and the current ripple migration rate, *u*_*r*_ (Van den Berg, 1987):

(13) $$q_{b}=0\cdot 6\left(1-P\right)u_{r}H$$

where *P* is the bed porosity. Equation (12) was expressed by Van Rijn (1993) also in terms of bed shear stress and sediment size:

(14) $$q_{b}=0\cdot 053\sqrt{\left(\rho_{s}/\rho \right)gD_{50}^{3}}D_{*}^{-0\cdot 3}{\left(\frac{\tau_{b}-\tau_{b,c}}{\tau_{b,c}}\right)}^{2\cdot 1}$$

where *τ*_*b*_ is the bed shear stress, and *τ*_*b,c*_ is the critical bed shear stress according to Soulsby (1997). Baas *et al*. (2000) found a power‐law relationship between flow forcing and ripple migration rate for Eq. (13), which in modified form can be written as:

(15) $$u_{r}=\alpha {\left(\theta^{\prime }-\theta_{c}\right)}^{\beta }$$

where *α* and *β* are coefficients dependent on sediment size.

Equations (12), (13), (14), (15) to (12), (13), (14), (15), as well as other bedload transport equations, have been used successfully for cohesionless sand, albeit with large degrees of uncertainty. The fact that: “it is hardly possible to predict the transport rate with an inaccuracy of less than a factor 2” (Van Rijn, 1993) might result in part from the lack of parameterisation of the effect of physical and biological cohesion in these equations. Even for quite modest amounts of clay and EPS, Lichtman *et al*. (2018) demonstrated large effects on the bed material transport rate associated with ripple migration. The study of Lichtman *et al*. (2018) and the present study, combined with the recent laboratory studies, on bedform development in mixed sand–clay–EPS (Baas *et al*., 2013; Malarkey *et al*., 2015; Parsons *et al*., 2016) suggest that predictions of *q*_*b*_ could be vastly improved if cohesive forces were included in bedload transport equations. Insufficient data are available at present to achieve this, but these improvements would require the modification of *τ*_*b,c*_ in Eq. (14) and *θ*_*c*_ in Eq. (15) for cohesive forces in the bed, even at several percent of clay and hundredths of a percent of EPS. An improved parameterisation of ripple height in Eq. (13) is also essential, because existing mathematical predictors for the size of current ripples (Raudkivi, 1997; Baas, 1999; Soulsby & Whitehouse, 2005), which relate the equilibrium height (and wavelength) of the ripples to the median sediment size within the ripples, have been developed only for non‐cohesive sand. Hence, these predictors are unlikely to be sufficiently accurate for mixed sand–clay–EPS.

The present study strengthens the evidence collected in previous studies (Baas, 1994, 1999) that current ripples are often smaller in size, and more two‐dimensional in planform, than those predicted from the size of the sand particles in the bed alone. In other words, the common presence of cohesive clay and EPS in the natural environment increases the likelihood of finding smaller ripples with non‐equilibrium planforms. By using an equation for the development of ripple height from a flat bed in clay‐free 0·228 mm sand (Baas, 1999), similar to Eqs (4) and (5) but with an initial height, *H*_0_, equal to zero, it can be shown that the smallest ripples in the study area were approximately 4 mm high, and the equilibrium height, *H*_*E*_, was 17 mm. For this 13 mm difference in ripple height alone Eq. (13) would overpredict the bedload transport rate by a factor of four, if the effect of cohesion is ignored. The presence of clay and EPS also increases *θ*_*c*_ which is likely to further worsen this overprediction (Fig. 7).

### Implications for geological studies

Whilst not a strong indicator of depositional environment, current ripples and their primary current lamination have been used extensively for the reconstruction of depositional processes in the geological record (e.g. Stow, 2005). The equilibrium height and wavelength of current ripples increase, as the median size of the sediment particles in the bed increases, but their equilibrium dimensions are independent of flow velocity (Baas, 1994). Instead, flow velocity accelerates the development towards equilibrium height and wavelength. On their way to linguoid equilibrium shape, current ripples go through distinct plan morphologies, i.e. incipient, straight‐crested, sinuous and non‐equilibrium linguoid (Baas, 1994). This progressive increase in three‐dimensionality has been used to determine the development stage of current ripples in deep‐marine and shallow‐marine environments (Baas, 1993; Oost & Baas, 1994). However, reconstructing flow properties, such as flow velocity and bed shear stress, from the size and shape of non‐equilibrium current ripples in clean sand has been challenging, because Eqs (4) and (5) show that the formation of non‐equilibrium ripples also includes a time factor. For example, the presence of underdeveloped straight‐crested ripples and associated tabular cross‐lamination may signify weak flow of relatively long duration, but also strong flow of very short duration. The present study shows that physical and biological cohesion push current ripples further away from 3D towards 2D shapes, unless the flow is strong enough to successfully winnow the clay and EPS from the bed. Above all, a full recognition of the importance of cohesive forces for bedform dynamics, even at low bed clay and EPS fractions, requires a paradigm shift in sedimentary facies analysis. Shepard (1954) put the boundary between clean sand and dirty sand at 25% clay, whereas Folk (1951) and Dott (1964) used 10 to 15% sediment <0·030 mm to distinguish between mature sandstone (‘arenite’) and immature sandstone (‘wacke’). These textural classifications have practical use for categorising rocks in outcrop and core, but the boundary percentages should not be used to differentiate non‐cohesive from cohesive dynamic behaviour of mixed sand–mud. Based on the laboratory and field data presented herein, a boundary between mature and immature sand of 3% detrital clay more accurately distinguishes non‐cohesive from cohesive mixed sand. This boundary limits the effect of cohesion to a 20% reduction in Λ (Fig. 6) and an 8% increase in *θ*_*c*_ (Fig. 7). At the study site, 3% detrital clay (Fig. 4A) was equivalent to 8% mud (<0·063 mm; Eq. (1)), a size fraction <0·030 mm of 7% and 0·06% EPS. Further work is needed to verify whether these boundaries are applicable beyond the limits of the present comparative study of laboratory and field bedforms.

As the development of current ripples in mixed sand–clay involves loss of clay and EPS by winnowing, facies analysis should not use the clay content within current ripples and ripple cross‐laminated sand to relate current ripple size and shape to bed cohesion and hydrodynamic forcing. However, the clay content in the sediment immediately below the base of current ripples and sets of cross‐lamination may in many cases be representative for the original bed clay content from which the ripples were generated, especially if the textural properties of the sediment – other than the clay content – are similar. On intertidal flats in estuaries, the field data show that time is an important limiting factor for the formation of current ripples. Following on from the above interpretations for mixed sand–clay in modern estuaries, the presence of 3D current ripples and trough cross‐lamination in intertidal sandstone with more than 13% clay requires extraordinary processes of formation, such as combinations of spring tides within the macrotidal range, promotion of bedload transport by near‐bed wave stress, close proximity to sandy tidal channels, and a large number of consecutive tidal cycles of slow ripple growth without destructive processes. Under normal circumstances, however, such clay‐rich sandstone is expected to be associated with a total absence of current ripples or with small, 2D, straight‐crested current ripples and tabular cross‐lamination, whereas fully developed linguoid current ripples and their cross‐lamination should dominate sandstone that is poor in cohesive clay. Based on the data presented (Figs 4 and 6), the 3% detrital clay defined above for the boundary between mature and immature sand in the subsurface might also be an appropriate upper boundary for the presence of 3D ripples and their cross‐lamination in sedimentary facies associated with tidal flats in macrotidal estuaries. This maximum in clay content is inferred to get progressively lower with decreasing tidal range in mesotidal and microtidal estuaries, with an increasing probability of finding sedimentary facies with 2D current ripples and their cross‐lamination or no ripples at all. Moreover, clay‐poor 3D current ripples are expected to be more common in tidal flat facies that are close to tidal channel facies, where tides accelerate owing to ebb and flood flow confinement.

## Conclusions

This study shows that laboratory experiments on current ripple development in mixed sand–clay–EPS are a suitable analogue for current ripple development on mixed sand–clay–EPS intertidal flats, and possibly also in other environments where physical and biological cohesion influence sediment erosion, transport and deposition. The comparison between the field and laboratory data has led to the following main conclusions:

1. Current ripples in sand develop at an increasingly slower rate, as progressively larger volumes of cohesive clay are mixed into the sand.
2. These current ripples tend to become smaller and change from three‐dimensional linguoid to two‐dimensional straight‐crested, as bed clay content is increased.
3. These findings demonstrate that clay starts to affect bedform dynamics in sandy sediment at much lower concentrations than for the ‘clean sand’ and ‘arenite’ of Shepard (1954) and Folk (1951). A revised boundary of 3% detrital clay, equivalent in the present experiments to 7% matrix sediment with a grain size <0·030 mm, appears more appropriate for the onset of bed stabilisation by cohesive forces in the seabed.
4. Extracellular polymeric substances (EPS) might have a similar, yet stronger, effect on the size and planform of current ripples than clay.
5. Clay type had a small influence on current ripple development in mixed sand–clay–EPS, because these bedforms were generated mostly at bed clay contents for which the rheological differences between clay mineral types are small.
6. Differences in cumulative flow forcing need to be accounted for in comparisons of the dynamics of current ripples in mixed sand–clay between field and laboratory.
7. Pore water salinity is also expected to control bedform development in mixed sand–clay–EPS, but quantification of this influence requires further study.
8. These findings have important implications for predicting current ripple properties from hydrodynamic data in hydraulic engineering and for reconstructing flow properties from current ripple size and shape in sedimentary geology.

## Figures and Tables

**Figure A1:**
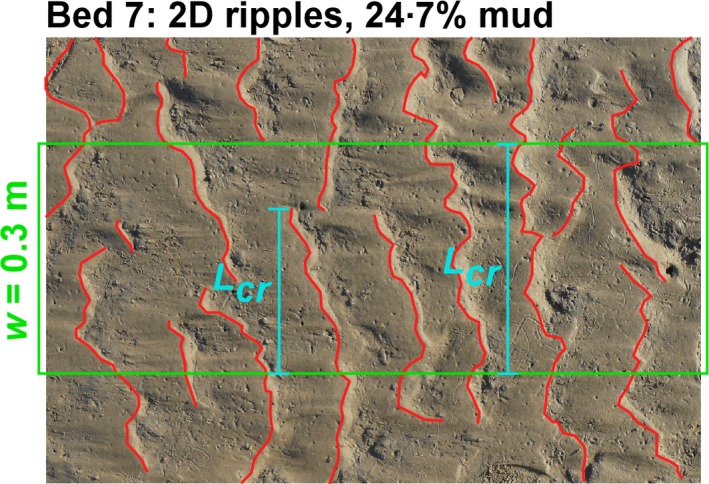
Summary of the methodology used to calculate ripple planform indices. A 0·3 m wide window was placed in the centre of the picture (in green), before tracing the ripple crestlines within this window (in red). Thereafter, the shortest distance, *L*_*cr*_, between the start and end of each crestline was measured (in blue) and divided by the width of the window to derive the ripple planform index as measure of ripple three‐dimensionality. Finally, the average value of *I* was calculated for each bed.

## Appendix A

### Calculation of Ripple Planform Indices

The visual descriptions of 2D, 2D–3D and 3D ripples in the field and in the experiments of Baas *et al*. (2013) were supported quantitatively by calculating the planform index, *I*, of the ripples:

(A1) $$I=L_{\mathrm{cr}}/w$$

where *L*_*cr*_ is the shortest distance between the start and end of the crestline of a ripple and *w* is a reference width, both perpendicular to the flow direction. Here, *w *=* *0·3 m, so that a direct comparison could be made between the data from the Dee Estuary and from Baas *et al*. (2013), who used a flume with a width of 0·3 m. The methodology is summarised in Fig. A1. Planform index values for the field and laboratory ripples are shown in Table 1 and Table A1, respectively. These data show that *I *>* *0·50 for 2D ripples, 0·39 ≤ *I *≤* *0·5 for 2D–3D ripples and *I *<* *0·39 for 3D ripples.

**Table A1:** Summary of morphological and textural data for selected experiments of Baas *et al*. (2013), including ripple planform indices

Run number	Mean ripple length (mm)	Ripple planform index	Bed clay %		Bedform type
			Initial	Ripple	
08	77·7	0·57	13·8	1·2	2D ripples
06	86·7	0·44	11·8	0	2D–3D ripples
04	98·4	0·33	5·4	0·3	3D ripples
02	95·5	0·31	1·8	0	3D ripples
01	116·3	0·28	0	0	3D ripples

## Appendix B

### Time for Complete Trapping of a Colloid

Following the approach of Malarkey *et al*. (2015), the time for complete trapping of a colloid, such as clay or EPS, as a result of Darcy's Law when ripples are stationary, can be estimated from fig. 5a of Packman *et al*. (2000). The threshold of motion according to their eqs (6), (7), (8), (9) to (6), (7), (8), (9) for an effective mean water depth of 1·73 m (fig. 5a) is *U *=* *0·361 m s^–1^. This value of *U* is approximately equivalent to 0·2 m s^–1^ for Packman *et al*.'s (2000) water depth and ripple size. According to their fig. 5a for *U *=* *0·2 m s^–1^, the time taken for the colloid concentration in the bed to reach 90% of its value in flow, *t*_90_, requires that *ku*_*m*_ *t*_90_/*P *=* *32·5. Here *k* is ripple wave number (=2π/*L*), *L* is the ripple wavelength, *u*_*m*_ is the maximum induced pore water velocity (=*kKh*_*m*_), *P* is the porosity (=0·4), *K* is the hydraulic conductivity (Hazen, 1911) and *h*_*m*_ is the half amplitude dynamic head (Packman *et al*., 2000), given by:

(B1) $$\begin{array}{cc} K∼10{D_{10}}^{2}, & h_{m}=0\cdot 14\frac{U^{2}}{g}{\left(\frac{H}{0\cdot 34d}\right)}^{3/8} \end{array}$$

where *K* is in mm s^–1^, *D*_10_ is in mm and *d* is the water depth. In this case, *D*_10_ = 0·149 mm, so that *K *≈* *0·22 mm s^–1^, *d *=* *1·73 m, *U *=* *0·2 m s^–1^, *H *≈* *0·1*L* and *L* is in the range 77 ≤ *L *≤* *143 mm. For these values, 19·7 ≤ *t*_90_ ≤ 61·7 h (0·8 to 2·6 days) or a minimum of four times the inundation period (4·7 h). The corresponding maximum pore water velocities are 0·08 ≤ *u*_*m*_ ≤ 0·13 mm min^–1^, which is two orders of magnitude smaller than the 6 mm min^–1^ bedform migration rate measured in the same area on 26 May 2013 (Lichtman *et al*., 2018). This demonstrates that winnowing via overturning dominated over pumping for this inundation period (Packman & Brooks, 2001). The timescale for the turnover of the ripples based on the migration rate and the range of ripple wavelengths is 0·2 to 0·4 h.

## Appendix C

### Description of Critical Shear Stress Experiments

The ‘vertical shear flume’ (Hydrodynamics Laboratory, Bangor University; McCarron *et al*., 2019) was used to estimate the critical shear stress for sediment motion from beds comprising mixed sand–clay–EPS (Fig. 7). This flume consists of a straight, rectangular channel, 1·2 m long and 0·18 m wide, with a water depth of 0·12 m, through which freshwater was recirculated by a variable‐speed pump. A block‐shaped sediment box was slotted onto the bottom of the channel, ensuring that the surface level of the sediment in the box was flush with the floor of the channel. The sediment column within the box was 0·14 m long, 0·1 m wide and 0·1 m deep. Downstream of this box, a bedload trap collected sediment particles moving along the bottom of the flume in flows that exceeded the critical shear stress for sediment motion.

The experiments were conducted with well‐sorted, fine sand (*D*_50_ = 0·142 mm) mixed with 0 to 1% xanthan gum (EPS proxy), 0 to 30% kaolin clay, and mixtures of up to 30% kaolin and up to 1% xanthan gum. In each experiment, steady, uniform flow was started well below the critical shear stress for the pure sand and then increased in small steps every 10 min. Streamwise flow velocities were measured with a vertical stack of five ultrasonic Doppler velocimetry (UVP) probes (Best *et al*., 2001). Bed shear stresses were calculated from the UVP data by squaring the friction velocity determined from standard logarithmic law of the wall techniques (Van Rijn, 1990). Water samples were taken periodically to determine suspended sediment concentrations, using the standard weighing and drying method, such that an optical backscatter (OBS) instrument, which was continuously recording, could be calibrated.

For each sand–clay–EPS mixture, the critical bed shear stress was determined by a combination of three methods: (i) visual observation of the erosion of sand grains from the sediment surface; (ii) initiation of infilling of the bedload trap; and (iii) the first significant increase in the concentration of suspended sediment. All critical bed shear stresses, relative to the critical bed shear stress for the pure sand, are shown in Fig. 7.
